# Polymorphism in *spa* gene of *Staphylococcus aureus* from bovine subclinical mastitis

**DOI:** 10.14202/vetworld.2016.421-424

**Published:** 2016-04-28

**Authors:** Taruna Bhati, Prerna Nathawat, Sandeep Kumar Sharma, Rahul Yadav, Jyoti Bishnoi, Anil Kumar Kataria

**Affiliations:** 1 Department of Veterinary Microbiology and Biotechnology, College of Veterinary and Animal Science, Rajasthan University of Veterinary and Animals Sciences, Bikaner, Rajasthan, India; 2 Department of Veterinary Microbiology and Biotechnology, Post Graduate Institute of Veterinary Education and Research, Rajasthan University of Veterinary and Animals Sciences, Bikaner, Rajasthan, India

**Keywords:** cattle, polymorphism, protein-A, *spa* gene, *Staphylococcus aureus*, subclinical mastitis

## Abstract

**Aim:**

The virulence-associated protein-A of Staphylococcus aureus, encoded by spa gene shows a variation in length in different strains. In this study, the spa gene variation in S. aureus strains was studied which were isolated from subclinical cases of bovine mastitis.

**Materials and Methods:**

About 38 isolatesof S. aureus were recovered from Holstein–Friesian (HF) crossbred (n=16) and Rathi cattle (n=22) with subclinical mastitis as per standard procedures, and these isolates were subjected to amplification of spa gene (X-region) by polymerase chain reaction and calculation of number of tandem repeats were done.

**Results:**

Of the 16 isolates from H-F crossbred cattle, all with the exception of one isolate produced spa amplicon. Seven isolates produced amplicons of 200 bp, one produced 160 bp, and other seven produced spa amplicon of 150 bp with calculated number of 6, 5, and 4 repeats, respectively, whereas nine different types of amplicons were produced by 22 S. aureus isolates from Rathi cattle, viz., 280, 250, 240, 200, 190, 180, 170, 150, and 140 bp with 10, 8, 8, 6, 6, 6, 5, 4, and 4 repeats, respectively. One of the isolates from Rathi cattle produced two spa amplicons (150 and 190 bp).

**Conclusion:**

A greater polymorphism was observed in the S. aureus isolates from Rathi cattle than from H-F crossbreds with subclinical mastitis.

## Introduction

Bovine mastitis is a well-known challenge to dairy industry in India. It affects the economy of farmers and hence of the country leading to an estimated annual loss of around US $526 million [1]. *Staphylococcus aureus* is the most important pathogen associated with various clinical forms of mastitis [2]. Among the various clinical forms of mastitis caused by *S. aureus*, subclinical cases have special importance as they go unnoticed and affect production performance of animal to a large extent [3].

The development and severity of mastitis depend on the production of virulent protein known as protein-A [4]. This protein is encoded by *spa* gene which has been shown to have a high degree of variability in size [5]. This variation in the *spa* gene comes from the differences in the repetitive variable number of 24 bp repeats in X-region of gene. The number of these 24 bp repeats varies among different strains of *S. aureus* and hence can be used as a molecular tool in studying the genetic diversity among the Indian strains of *S. aureus* for epidemiological tracing of source of infection and comparing the differences in virulent phenotypes among various strains. Although a lot of work has been conducted in typing of *S. aureus* from human cases in India [6-8], very limited work has been done in studying the genetic diversity using *spa* gene of *S. aureus* strains originating from bovine mastitis [9].

In view of the above facts, the present investigation was designed to study the polymorphism of *spa* gene (X-region) and evaluate its applicability in differentiating the Indian *S. aureus* strains of bovine origin.

## Materials and Methods

### Ethical approval

This study was conducted following approval by the research committee and Institutional Animal Ethics Committee Guidelines were followed.

### Isolation of *S. aureus*

#### Sampling

Eighty-five milk samples were collected during early morning hours in sterilized test tubes from Holstein–Friesian (H-F) crossbred and Rathi cattle from different locations in Bikaner (Rajasthan, India). The samples were immediately taken to the laboratory for further processing on ice.

### Somatic cell counting (SCC)

A 0.1 ml amount from each properly shaken milk samples was withdrawn with Pasteur Pipette and spread evenly on a glass slide to count the SCC as per the method described earlier [10].

### Identification of *S. aureus*

All the milk samples which showed SCC corresponding to subclinical mastitis were processed for isolation of *S. aureus*. Phenotypic and biochemical identification of isolates were done as per the standard protocol [11]. The isolates were further genotypically confirmed by *23S rRNA* species-specific polymerase chain reaction (PCR) using forward primer-1 (5’-ACGGAGTTACAAAGGACGAC-3’) and reverse primer-2 (5’-AGCTCAGCCTTAACGAGTAC-3’) [12].

### Amplification of spa gene

The amplification of *spa* gene encoding protein-A was done as described by Frenay *et al*. [13] with slight modifications using 5’-CAAGCACCAAAAGAGGAA-3’ (F) and 5’-CACCAGGTTTAACGACAT-3’ (R) primers. PCR was performed in 0.2 ml thin-walled PCR tubes. The PCR mixture contained a final concentration of 10 mM Tris–HCl, pH 9.0, 50 mM KCl, 3.5 mM MgCl_2_, 1.0 μM concentration of each primer, 0.2 mM concentrations of each 2’-deoxynucleoside 5’-triphosphate and 1.0 U of Taq DNA polymerase. The PCR was performed in Palmcycler (Corbett Research, Australia) using following cycling parameters: Initial 34 cycle of amplification (denaturation at 94°C for 60 s, primer annealing at 55°C for 60 s and primer extension at 70°C for 60 s), and final extension at 72°C for 5 min. Two μl of trekking dye was added to the PCR products and were resolved in 1.2% agarose gels prepared in 1× TBE buffer containing 0.5 μg/ml of ethidium bromide. 100 bp DNA ladder was used as molecular marker and the amplification products electrophoresed for 1 h at 100 V. The gel was then visualized under U.V. Transillumination and photographed. Calculation of a number of tandem repeats (N) in PCR amplified *spa* gene product was done using the formula given by Frenay *et al*. [13]. Mathematically, formula is given as:

## Results

Out of the 85 milk samples, 38 milk samples showed SCC in the range of 200×10^3^ to 500×10^3^ cells/ml corresponding to subclinical cases of mastitis as per the IDF (2005) criterion [14]. The SCC has been detected to be the most reliable test and closest to the bacteriological results for SCM in dairy cows by Sharma *et al*. (2010) [15]. A total of 38 isolates of *S. aureus* were isolated from these samples and identified on the basis of cultural and biochemical properties. All 38 isolates producedan amplicon of 1,250 bp in species-specific PCR targeting *23S rRNA* gene. Out of 38 isolates, 16 were isolated from H-F crossbred cattle and 22 from native Rathi cattle.

**Figure 1:**
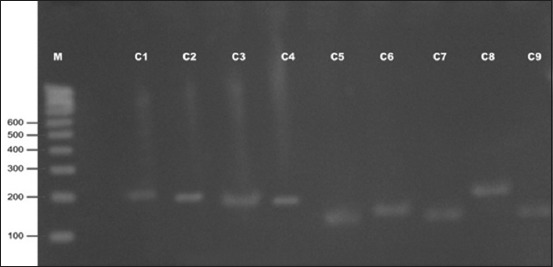
Polymerase chain reaction amplicons of spa gene (X-region) of Staphylococcus aureus isolates from Holstein–Friesian crossbred cattle (C1-C9) with subclinical mastitis.

**Figure 2:**
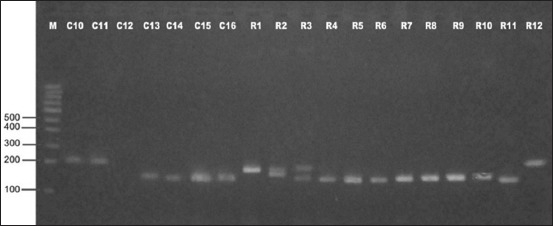
Polymerase chain reaction amplicons of spa gene (X-region) of Staphylococcus aureus isolates from Holstein–Friesian crossbred cattle (C10-C16) and Rathi cattle (R1-R12) with subclinical mastitis.

**Figure 3:**
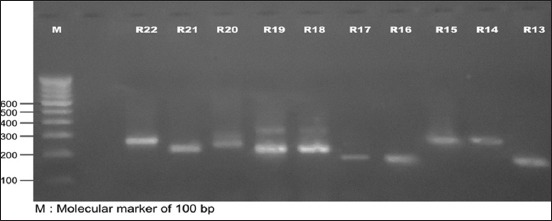
Polymerase chain reaction amplicons of spa gene (X-region) of Staphylococcus aureus isolates from Rathi cattle (R13-R22) with subclinical mastitis.

In the present investigation, out of 16 isolates from H-F crossbred cattle, 15 strains produced *spa* amplicons, whereas one isolate did not produce any amplified product (Figures-1 and 2). Seven isolates produced amplicons of 200 bp, one produced 160 bp amplicon, and other seven produced amplicon of 150 bp with calculated number of 6, 5, 4 repeats, respectively (Table-1). The *spa* gene X-region amplicons produced by 22 isolates from Rathi cattle were of greater variability (Figures-2 and 3) than that in isolates from H-F crossbred cattle as nine different types of amplicons were obtained of size 280, 250, 240, 200, 190, 180, 170, 150, and 140 bp with calculated number of 10, 8, 8, 6, 6, 6, 5, 4, and 4 repeats, respectively (Table-2). The amplicon of 150 bp size was found to be produced by maximum (15 isolates) number of isolates followed by amplicons of 200 bp (11 isolates) and 280 and 240 bp (three each). One isolate from Rathi cattle produced two bands of *spa* amplicons (150 and 190 bp).

**Table-1:** *spa* gene (Xregion) polymorphism in *S. aureus* isolates from HF crossbred cattle with subclinical mastitis.

Serial number	Isolate numbers	Total isolates	*spa* gene amplicon (bp)	Total number of repeats
1	C1, C2, C3, C4, C8, C10, C11	7	200	6
2	C6	1	160	5
3	C5, C7, C9, C13, C14, C15, C16	7	150	4

*S. aureus=Staphylococcus aureus*, HF=Holstein–Friesian

**Table-2:** *spa* gene (X-region) polymorphism in *S. aureus* isolates from Rathi cattle with subclinical mastitis.

Serial number	Isolate numbers	Total isolates	*spa* gene amplicon (bp)	Total number of repeats
1	R14, R15, R22	3	280	10
2	R20	1	250	8
3	R18, R19, R21	3	240	8
4	R12, R13, R16, R17	4	200	6
5	R3	1	190,150	6,4
6	R1	1	180	6
7	R2	1	170	5
8	R4, R5, R6, R7, R8, R9, R10	7	150	4
9	R11	1	140	4

S. aureus=Staphylococcus aureus

## Discussion

The PCR amplification of *spa* gene (X-region) yielded amplicons similar to that recorded by Salasia *et al*. [16] who obtained nine different sized amplicons of 100-340 bp in *S. aureus* isolates from bovine subclinical mastitis. Bystron *et al*. [17] also recorded 10 different sizes of *spa* amplicons in the *S. aureus* isolates from unprocessed cow milk, but their amplicon size varied from 3 to 14 repeats having the highest frequency of eight to 10 repeats. In our study, however, the size varied from 4 to 10 repeats with a maximum frequency of four repeats.

The *spa* types in this study corroborated the earlier observations of Karahan *et al*. [18] who also carried out *spa* typing of *S. aureus* strains isolated from bovine subclinical mastitis and recorded nine *spa* types with amplicons ranging from 100 to 320 bp where most of the *spa* types were similar to that obtained in this study. However, contrarily, they obtained *spa* amplicons with 290 bp and 10 repeat units as predominant *spa* type, whereas in our study 150 bp *spa* amplicons with four repeats were predominant.

In our study, only seven of the isolates produced *spa* amplicons with calculated number of more than seven repeats. Freney *et al*. [19] reported that most epidemic MRSA strains harbored more than seven repeats while non-epidemic MRSA strains contained seven or fewer repeats. They discussed that a longer X-region results in a better exposition of the Fc binding region of protein-A thereby facilitating colonization on both surfaces and contributing to the epidemic phenotypes. Considering the above fact, in the present investigation less number of isolates were detected to be pathogenic in regards to *spa* typing.

One isolate from Rathi cattle produced two bands of *spa* amplicons (150 and 190 bp) which are in conformity to the earlier observation by Rathore *et al*. [20] who recorded two *spa* bands in one isolate of *S. aureus* isolated from camel skin wounds. One of the 38 isolates did not produce *spa* amplicon. The absence of *spa*-X region gene has also been reported by Kalorey *et al*. [21] in subclinical mastitis, Momtaz *et al*. [22] from bovine clinical and subclinical mastitis, Salem-Bekhit *et al*. [23] in bovine mastitis isolates, and Shakeri *et al*. [24] in healthy carriers and human patients.

## Conclusion

This study revealed polymorphism in *spa* X-region gene amplicons of *S. aureus* obtained from subclinical mastitis cases. A greater polymorphism was observed in the isolates from native breed. Based on the number of repeats, it was deduced that in this study though both pathogenic and non-pathogenic strains were recovered from sub-clinical mastitis cases but nonpathogenic strains were more in number.

## Authors’ Contributions

AKK was the major guide of my MVSc research work and he planned and designed the study. This work is a part of my MVSc thesis. RY and PN helped in conducting the Laboratory work. Lab analysis was carried out by SKS and JB. The manuscript was revised and edited under the guidance of AKK. All authors participated in writing and revision process and approved of the final manuscript.
